# Mouse Genome Database: From sequence to phenotypes and disease models

**DOI:** 10.1002/dvg.22874

**Published:** 2015-07-27

**Authors:** Janan T. Eppig, Joel E. Richardson, James A. Kadin, Cynthia L. Smith, Judith A. Blake, Carol J. Bult, the MGD Team

**Affiliations:** ^1^ The Jackson LaboratoryBar HarborMaine

**Keywords:** mouse, database, genomics, gene function, human disease models

## Abstract

The Mouse Genome Database (MGD, www.informatics.jax.org) is the international scientific database for genetic, genomic, and biological data on the laboratory mouse to support the research requirements of the biomedical community. To accomplish this goal, MGD provides broad data coverage, serves as the authoritative standard for mouse nomenclature for genes, mutants, and strains, and curates and integrates many types of data from literature and electronic sources. Among the key data sets MGD supports are: the complete catalog of mouse genes and genome features, comparative homology data for mouse and vertebrate genes, the authoritative set of Gene Ontology (GO) annotations for mouse gene functions, a comprehensive catalog of mouse mutations and their phenotypes, and a curated compendium of mouse models of human diseases. Here, we describe the data acquisition process, specifics about MGD's key data areas, methods to access and query MGD data, and outreach and user help facilities. genesis 53:458–473, 2015. © 2015 The Authors. Genesis Published by Wiley Periodicals, Inc.

## INTRODUCTION

The laboratory mouse is widely recognized as an important animal model for investigating genetic and cellular systems relevant to human biology and disease. It has the advantages of being a small mammal in which all life stages can be accessed, for which a completely sequenced, well annotated reference genome is publicly available, and for which there are myriad genomic tools available for comparative and experimental manipulation. These include thousands of unique inbred strains listed in the International Mouse Strain Resource (www.findmice.org), which catalogs resources available in pubic repositories (currently over 32,000 strains and more than 200,000 embyronic stem (ES) cell lines).

In addition to the mouse reference genome sequence from the C57BL/6J inbred strain, 17 additional strains have been deeply sequenced (Keane *et al*., 2011), with sequence for an additional 11 inbred strains available, but not yet published (K. Hunter and T.M. Keane, see http://www.sanger.ac.uk/resources/mouse/genomes/). For many strains, extensive single nucleotide polymorphism (SNP) data exist (e.g., dbSNP, Sayers *et al*., 2012; Center for Genome Dynamics SNP data http://cgd.jax.org/cgdsnpdb/; Sanger Mouse Genomes Project, http://www.sanger.ac.uk/resources/mouse/genomes/), as well as data on genome structural variation (Yalcin *et al*., 2012).

A number of large‐scale mouse mutagenesis programs have created ready access to pre‐generated, preserved mutations on defined genetic backgrounds. These include the International Gene Trap Consortium, producing ≈190,000 gene‐trapped ES cell lines (Nord *et al*.,^2006^, ^1^), a number of *N*‐ethyl‐*N*‐nitrosourea (ENU)‐mutagenesis projects producing over 3,300 defined mutations (e.g., see Andrews *et al*., 2012; Boles *et al*., 2009; Clark *et al*., 2004), and the International Knockout Mouse Consortium (IKMC) generating targeted mutations in all mouse protein‐coding genes in ES cell lines, with over 14,000 genes successfully targeted to date (Bradley *et al*., 2012). Genetic engineering methodologies including CRISPR/Cas (Clustered Regularly Interspaced Short Palindromic Repeats/CRISPR associated sequences) engineering (Wang *et al*., 2013) and elegant, technologies that allow spatiotemporal control of gene ablation and regulation are providing new insight into gene function through the use of conditional mutagenesis (Murray *et al*., 2012). Other new resources for complex traits and QTL (Quantitative Trait Loci), including the Collaborative Cross (Collaborative Cross Consortium, 2012; Threadgill and Churchill, 2012) and Diversity Outbred mice (Churchill *et al*., 2012; Svenson *et al*., 2012) are beginning to bear fruit in analysis of complex and multigenic diseases (Chesler, 2014; Logan *et al*., 2013; Philip *et al*., 2011).

These exquisite genetic resources for mice help unravel the functional components of the mouse (and mammalian) genomes, at the level of the individual gene, complex interactions, and systemic effects (Bucan *et al*., 2012). The large‐scale efforts of the International Phenotyping Consortium (Brown and Moore, 2012), with a goal to systematically phenotype 20,000 mouse lines developed from the IKMC knockout ES cell line resource, are currently producing a global sweep of high throughput phenotyping data that add new breadth to the careful, detailed phenotyping done by hundreds of researchers worldwide and captured in the MGD.

The Mouse Genome Database (MGD) has, over the course of its 25+ year history, moved from linkage maps to sequence maps, from visual to molecular phenotypes, and from small data (single genes, biochemical variation) to genome size data (gene sets, comparative phenotypes, genome‐wide variants), adapting to the changing scientific landscape and the improvements in computation and information technology. This article provides an overview of MGD. First, we describe the underlying requirements for data standards and integration that facilitate robust data retrieval and the process of data acquisition in MGD. This is followed by specifics about the breadth and depth of data content found in MGD and the multiple ways users can access and query these data and work with data sets, including through the web interface and customized searches. Next, a section is devoted to MGD's community interactions that supply documentation, online help, workshops, and ongoing communication to users; as well as receiving from users input on MGD's data, access and tools, and new research data submissions. Finally, we comment on the ongoing changes and challenges of maintaining and evolving MGD as an international community data resource for mouse genomics and biology.

## DATA INTEGRATION AND THE ROLE OF SEMANTIC AND DATA STANDARDS

**Figure 1:**
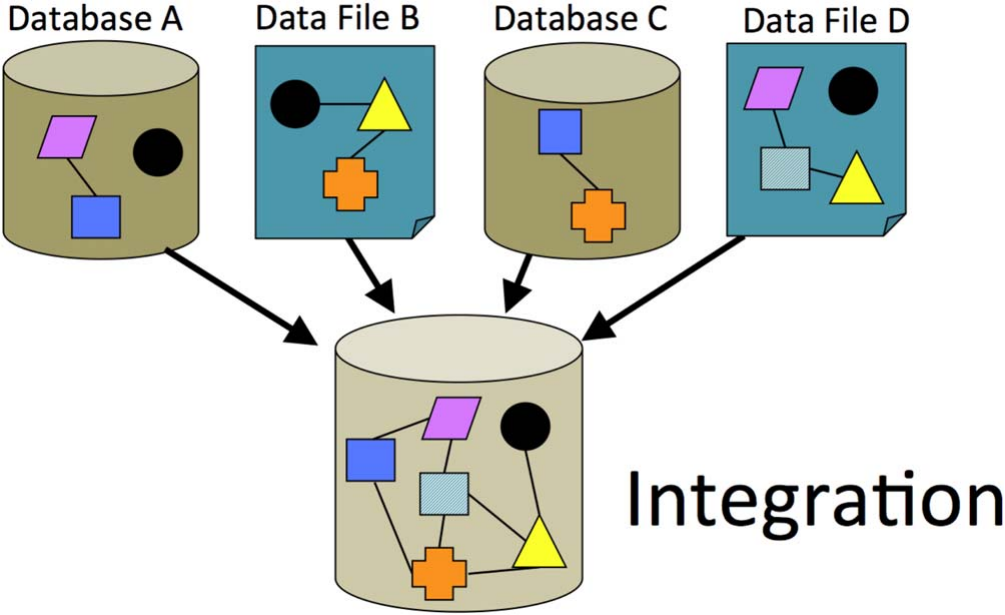
Data integration. The process of data integration includes: (**a**) gathering data from various sources (other database resources, electronic files from online submissions or literature curation, etc.), (**b**) identifying common objects among the input, (**c**) resolving conflicts or inconsistent information or discovering missing data, and (**d**) assembling the relationships among the resolved data. Integration is key to knowledge discovery. For example, in this diagram the integrated data of the pink object is now seen to have a relationship to the orange object via its association with the dark blue square and light blue square, although the pink and orange objects were not coincident in the incoming data.

Data integration is an underlying principle for MGD. To truly utilize the power of the mouse as a model system, disparate data must be brought into a common framework. The integration of data from many sources requires applying common semantic standards across those data. This process adds value to the data and creates a true knowledgebase, where new biological insights can emerge from novel data connections based on common, shared, identified objects (Fig. 1).

### Nomenclatures and Biological Ontologies

Human language is rich with descriptors and alternate ways of expressing the same or similar concepts. For scientific discourse, however, use of common terminology is essential to ensure that identical entities are clearly delineated. Unique identifiers (accession IDs) are used successfully in databases for this purpose, but generally human discussion relies upon words and symbols. This fact (along with vagaries of manuscript formats) has made precision in attempts at automating data extraction from the scientific literature via Natural Language Processing (NLP) difficult and further emphasizes the necessity for standard nomenclatures, ontologies, and the addition of consistent metadata to biological data sets and publications.

A simple example illustrates the value of using standard nomenclatures. Suppose an investigator is interested in finding information about the mouse gene *Tap*. The problem is that five different mouse genes have been published using this gene symbol. Fortunately for this investigator, the non‐standard uses of this symbol have been captured by MGD as synonyms to the correct gene nomenclature and all of these genes will be returned from doing a search^2^ for *Tap*. The user will still need to examine the gene alternatives to determine which is his/her actual gene of interest among those called *Tap* in the literature (in this case, each is on a unique chromosome, which might be enough of a clue). Two key points are: (1) that the investigator might have pursued a disastrous path by basing new experiments on data that was not about the gene he/she thought it was, or developed an erroneous view of *Tap* function that combined information about multiple genes had he/she not taken advantage of MGD's nomenclature curation; and (2) conversely, using non‐standard nomenclature in publications may result in one's work not being recognized or misinterpreted. MGD is the authoritative source for nomenclatures for genes, genome features, mutations, alleles, structural variants, and mouse strains. Nomenclature guidelines are available at: www.informatics.jax.org/nomen and assistance with new nomenclature is available by emailing nomen@jax.org.

Biological ontologies go beyond naming objects. Ontologies are structured vocabularies and the placement of terms within that structure relay information about the relationships of terms to one another and the type of relationship involved. Within MGD, the Gene Ontology (Gene Ontology Consortium, 2000) and the Mammalian Phenotype Ontology (Smith *et al*., 2005) are used extensively for function and phenotype annotations, respectively. In addition, the EMAP/EMAPA (Edinburgh Mouse Atlas Project/Edinburgh Mouse Atlas Project Abstract version) ontology of mouse developmental anatomy (Finger *et al*., 2015; Hayamizu *et al*., 2013) is used to coordinate anatomical term use among the ontologies.

The Gene Ontology (GO) was initiated over 15 years ago by MGD, the *Saccharomyces* Genome Database (SGD), and *Drosophila* Database (Flybase) (GO Consortium, 2000) and is composed of three distinct ontologies, for molecular function, biological process, and cellular component. GO has been widely adopted and applied to gene and gene product annotations in hosts of organism databases and is commonly used for enrichment analyses of large data sets (Gene Ontology Consortium, 2015). In MGD, GO is used to annotate mouse gene and gene product functions.

MGD began developing the Mammalian Phenotype Ontology (MP) in 2003 as a replacement for textual descriptions of mouse mutants, which were becoming too numerous to properly maintain and effectively search. The MP Ontology has also been adopted by the Rat Genome Database (RGD, www.rgd.mcw.edu), the Medical Research Council, Harwell, UK, the Sanger Institute, UK, and a number of large‐scale mutagenesis centers such as the ENU mutagenesis groups (e.g., see Ayadi *et al*., 2012; Goldowitz *et al*., 2004; Morgan *et al*., 2010), and the International Mouse Phenotyping Consortium (IMPC) that is phenotyping the targeted knockouts developed by the IKMC (Koscielny *et al*., 2014).

Use of standard semantics via nomenclatures and ontologies enables wide dissemination of scientific results and ready integration with other data applying these same standards. In the end, this standardization greatly benefits the community, enabling comprehensive searches in the data space and producing better returns with more complete results.

## DATA ACQUISITION: COMBINING AUTOMATION AND CURATION

The size and complexity of MGD data has grown tremendously since its inception, as has the ability of the scientific community to generate larger and larger data sets at a rapid pace. MGD has responded by continually improving its processes, automating data acquisition where feasible, developing semi‐automated curation and data verification tools, and streamlining curation procedures. Data submissions directly from large‐scale projects and individual laboratories are encouraged (see Community: Outreach and User Support section below).

Today, the bulk of MGD data are loaded and integrated automatically from over 50 external sources. Additional data are loaded from files prepared by MGD data analysts or are data relationships computed internally by MGD. In some cases, MGD loads are required to create new database objects and relationships. These loads, which occur weekly, include quality control checks that generate reports with any anomalies found. These reports are reviewed and anomalies resolved (either with the data provider or via curator analysis) as part of the curation and standardization process for MGD.

Critically important data continue to be published in peer‐reviewed journals, with more detail and specificity than those produced in large‐scale projects and of particular significance in validating large‐scale data inferences. The key for MGD is to identify, select, and curate the set of publications providing the highest quality data for new biological understanding and providing the best new information to enable hypothesis generation and relevance for human health.

Scientific curators and analysts identify and prioritize newly published articles for inclusion in MGD based on novelty (e.g., new mutant allele created, new gene function described) and whether a model for human disease is included. Articles are tagged with the genes and mutant alleles described, ensuring that nomenclature is correct. In the last 5‐year period, the average number of new publications indexed and/or curated in MGD is ∼12,000 each year.

Prioritization and full curation of articles follow different strategies, depending on article content. For example, for GO annotations, the highest priority is to assign gene or gene‐product function to those genes that have no previously known function or function has only been inferred by sequence similarity to another species. In contrast, new disease models and new or extended phenotyping data are high priority for capturing phenotypic manifestations.

## KEY DATA CLASSES IN MGD

### The Unified Gene Catalog

MGD produces and maintains a current unified, non‐redundant catalog of mouse genes and genome features, including, but not limited to, protein‐coding genes, pseudogenes, non‐coding RNA genes, quantitative trait loci (QTL), and heritable phenotypic markers. The gene catalog's foundation is the unified set of genome annotations resulting from the most recent assembly of the mouse reference sequence (C57BL/6J) as provided by NCBI (Sayers *et al*., 2012), Ensembl (Flicek *et al*., 2013 ), and Havana/Vega (Wilming *et al*., 2008). We use the fjoin (feature join) algorithm developed by Richardson (2006) at MGD to determine the equivalency of predicted genes by examining genome coordinate overlap of exons. Where gene predictions are non‐conflicting (i.e., either a genome feature in one data set overlaps only one feature in another data set or a gene prediction is unique to a data set) those features are loaded into MGD. Where complex overlap relationships are detected (i.e., a genome feature in one data set overlaps more than one feature in another data set (1:*n* or *n*:1); or multiple such overlaps are detected (*n*:*m*), quality control (QC) reports are generated and inspection of the data by a curator and communication with the data providers follows to reconcile the data. Updates are processed periodically as released by the annotation providers. Ongoing analysis of conflicting data is done in collaboration with the Mouse Genome Annotation group consisting of curators and analysts from MGD, Ensembl, NCBI, Havana/Vega, and the UCSC (University of California, Santa Cruz) browser team.

### Functional Annotations Using GO

MGD is one of the founding groups of the Gene Ontology Consortium (Gene Ontology Consortium, 2000). MGD is a major contributor to the ongoing development and refinement of the Ontology itself, in both its terminology base and structure. MGD is the authoritative source for GO functional annotations of mouse protein‐coding and functional RNA genes. Mouse GO annotations are derived by curation of the scientific literature, through inferences based on orthology from experimental data in other organisms, notably human and rat (Reference Genome Group of the Gene Ontology Consortium, 2009), and through inferences based on protein structural domains (Mitchell *et al*., 2015). MGD's mouse GO annotations are contributed to the GO Consortium Gene Ontology database, resulting in their wide distribution to other resources and use in large‐scale functional analyses.

### Genetic Mutations and Variants

MGD maintains a comprehensive catalog of mouse mutations that have arisen spontaneously or have been induced by mutagens (e.g., chemical, radiation) or created through genetic engineering. For large mutagenesis projects, MGD develops project‐specific loads which define the nomenclature that needs to be created, as well as a standard mutation creation statement utilizing appropriate parameter for each new mutant allele (e.g., genomic insertion site, transgene construct characteristics), strain on which the mutation is developed and/or bred, etc. Load processes include running scripts that generate QC reports from load log files to aid curators in identifying, communicating, and resolving data issues with the providers.

Despite significant large‐scale mutation‐generating projects, individual investigators continue to create many of their own new mouse mutations. This is no surprise, as large‐scale projects tend to create new mutations in many genes, but all are of the same type and for in‐depth studies for the discovery of gene function, genetic interactions, and strain‐specific modifier or epigenetic effects, very specific mutations (or sets of mutations) are required. Thus, although MGD has added thousands of mutations from large‐scale mutagenesis programs, 150–200 new mutant alleles are incorporated from individual laboratories each month through a combination of literature curation and direct online submission from investigators. (See User Input and Data Submission section below.)

### Phenotypes

MGD captures phenotypes from defined genotypes carried on various strain backgrounds using the Mammalian Phenotype (MP) Ontology (Smith and Eppig, 2012). Each genotype, described by a set of one or more mutant alleles in one or more genes, and carried in a specific strains background, can display an array of different phenotypes. For example, mutations in the gene *Kit* (kit oncogene), which has had considerable study, illustrate the large combinatory explosion that can occur with phenotypic data. *Kit* has 153 mutant alleles documented in MGD, 112 of which have been reported with one or more distinct “genome‐type” (different allelic combinations and/or different strain backgrounds and/or a compound genotype with multiple mutant genes). An additional 41 alleles of *Kit* are gene trapped or from IKMC and only exist in ES cell lines. As of early May 2015, for all genes, MGD includes 54,909 distinct “genome‐types” with 276,712 MP annotations, and these phenotype annotations include mutant alleles for 10,955 genes.

The Mammalian Phenotype (MP) Ontology is essential for the curation of phenotype data. It is a structured vocabulary whose initial development and continued maintenance is centered at MGD. Building and maintaining the MP Ontology is a dynamic process. New terms are added to the MP Ontology as curators require new terms (usually more granular terms) to annotate phenotypic studies. Suggestions for new terms, definitions, synonyms, and changes in ontology organization are tracked in SourceForge, (http://sourceforge.net/p/obo/mammalian-phenotype-requests/). In addition, work on particular branches of the MP Ontology has been spurred by the need for outside projects to use the MP Ontology, (e.g., we are working with the Cardiovascular Development Consortium (CvDC) funded by NHLBI's Bench to Bassinet program which is, in part, creating new mouse ENU mutations for congenital heart defects, Li *et al*., 2015). New terms are being added to the MP Ontology to complete and harmonize congenital heart defect terms with those used in pediatric clinics. As of early May 2015, the MP Ontology contained more than 10,900 phenotypic terms.

The MP Ontology can support general and granular phenotype knowledge and allows robust searching and retrieval for web‐based users as well as computational users. Each vocabulary term has an accession identifier, term name, synonyms, definition, and a reference for the definition. Terms are placed within a DAG (directed acyclic graph) vocabulary structure with relationships between parent and child nodes. Terms are organized from general to specific, allowing phenotypes to be annotated at the finest level of granularity possible to reflect current knowledge.

### Mouse Models for Human Disease

MGD curates the association between mutant mouse genotype/strain combinations (genome‐type) and human diseases, where those mouse genotypes are experimentally determined models of human diseases. This provides an accurate representation of the disease model, as just as in humans, the phenotypic manifestation of a disease‐causing mutation may differ significantly depending on the composition of the genetic background that may include modifying loci.

Annotations to human disease are based on published experimental assertions using human disease terms from OMIM (Online Mendelian Inheritance in Man, http://omim.org). There are five types of mouse models to human disease relationships used by MGD: models with phenotypic similarity to human disease where the causative gene in both the human disease and the mouse model involves mutations in orthologous genes; models with phenotypic similarity to human disease, but where the etiologies are distinct (mutations are not in orthologous genes); models with phenotypic similarity to human disease, but where the etiology is unknown; models with phenotypic similarity to human disease, and the mouse carries a transgene insertion (sometimes of a mutant human gene); and “not” models, where phenotypic similarity to the human disease was expected (usually based on creating a knockout of the human ortholog), but the resulting mice did not display symptoms of the human disease. As of May 2015, more than 4,530 mouse genome‐types have been associated with at least one OMIM disease, covering over 1,350 human diseases. See the section below on the Human‐Mouse: Disease Connection interface that can be used to examine both experimentally supported mouse models, as well as suggest candidates for mouse models, and potential human gene candidates based on mouse models.

## WEB INTERFACE ACCESS TO MGD

MGD provides an extensive set of search forms, browsing tools, pre‐generated downloadable data files, analysis tools, developer tools, various informational pages, and numerous links to other resources. A guide to several common ways to search MGD and the data that can be retrieved using those methods can be found in Table 1.

**Table 1:** Common Ways to Search MGD and the Data That Can Be Retrieved^a^

Search form	Access from	What is searched for	What is retrieved
Quick Search (most used access method)	Located prominently at the top of the topics index boxes on the homepage (www.informatics.jax.org) and in the upper right corner of other web pages	Broad searches through all MGD data, including nomenclature, vocabulary/ontology terms, and annotations; provides less search specificity, but greater breadth in a simple search	Genome Features (protein coding genes, non‐coding RNA genes, QTL, symbol synonyms, etc.) and Vocabulary Terms (GO terms, Phenotype terms, Diseases, etc.) with links to data details
Genes and Markers Query	Use Search pull‐down on the navigation bar (Genes) or select Genes from topic index boxes on the homepage	Genes and genome features using search parameters: nomenclature, feature type (*e.g*., protein coding gene), genome location, InterPro Domains, GO, Phenotypes, or Diseases annotations	Genes and genome features with location, maps, homologs, mutants and alleles, GO annotations, embryonic expression, sequence and protein links, references
Phenotypes, Alleles & Disease Model Search	Use Search pull‐down on the navigation bar (Phenotypes) or select Phenotypes & Mutant Alleles from topic index boxes on the homepage	Mutant or genetically engineered alleles, transgenes, QTL, etc. using search parameters: phenotype or disease terms, gene or allele nomenclature, genome location, allele generation method and/or allele attributes, or allele project collections	Summary of alleles for specified parameters and displaying allele attributes, system level phenotypes, and human diseases modeled. Links to genotypes, phenotypes, and disease annotations
Human‐Mouse Disease Connection	Use Search pull‐down on the navigation bar (Human Disease) or select Human‐Mouse: Disease Connection from the topic index boxes on the homepage	Mouse or human genes and orthologs, genome locations, phenotypes and disease terms. Searches accept mouse or human values as input; VCF files and text files of genes or IDs are also accepted input	Grid and table views showing mouse/human orthologs, phenotype classes, and human diseases/disease models fitting the parameters entered
Mouse Genome Browser	Use Search pull‐down on the navigation bar (Mouse Genome Browser) or select the Browser under the Genes topic from the homepage	Search by chromosome and genome coordinates	Optionally turn on tracks for mutant alleles, SNPs, QTL, phenotypes, or switch to viewing human GRCh38 build or pseudo genomes for strains other than the C57BL/6J reference genome
Vocabulary Browsers	Use Search pull‐down on the navigation bar and, hovering over the Vocabularies section, select GO, Mammalian Phenotype Ontology, OMIM	Search or browse vocabulary terms and select term of interest; GO and Phenotype Ontologies terms are displayed hierarchically; OMIM terms are displayed alphabetically	GO and MP terms provide definitions, synonyms and links to all annotations for the selected term; OMIM terms link to MGD Disease Model pages and to OMIM entries

a This is not an exhaustive list of search methods or data that can be retrieved from MGD. Users are encouraged to explore MGD, visit the “Getting Started” section on the homepage (www.informatics.jax.org), the Help, and FAQ sections in the upper left of each web page, and to contact User Support (email mgi-help@jax.org) for additional questions and assistance.

### MGD Homepage

**Figure 2:**
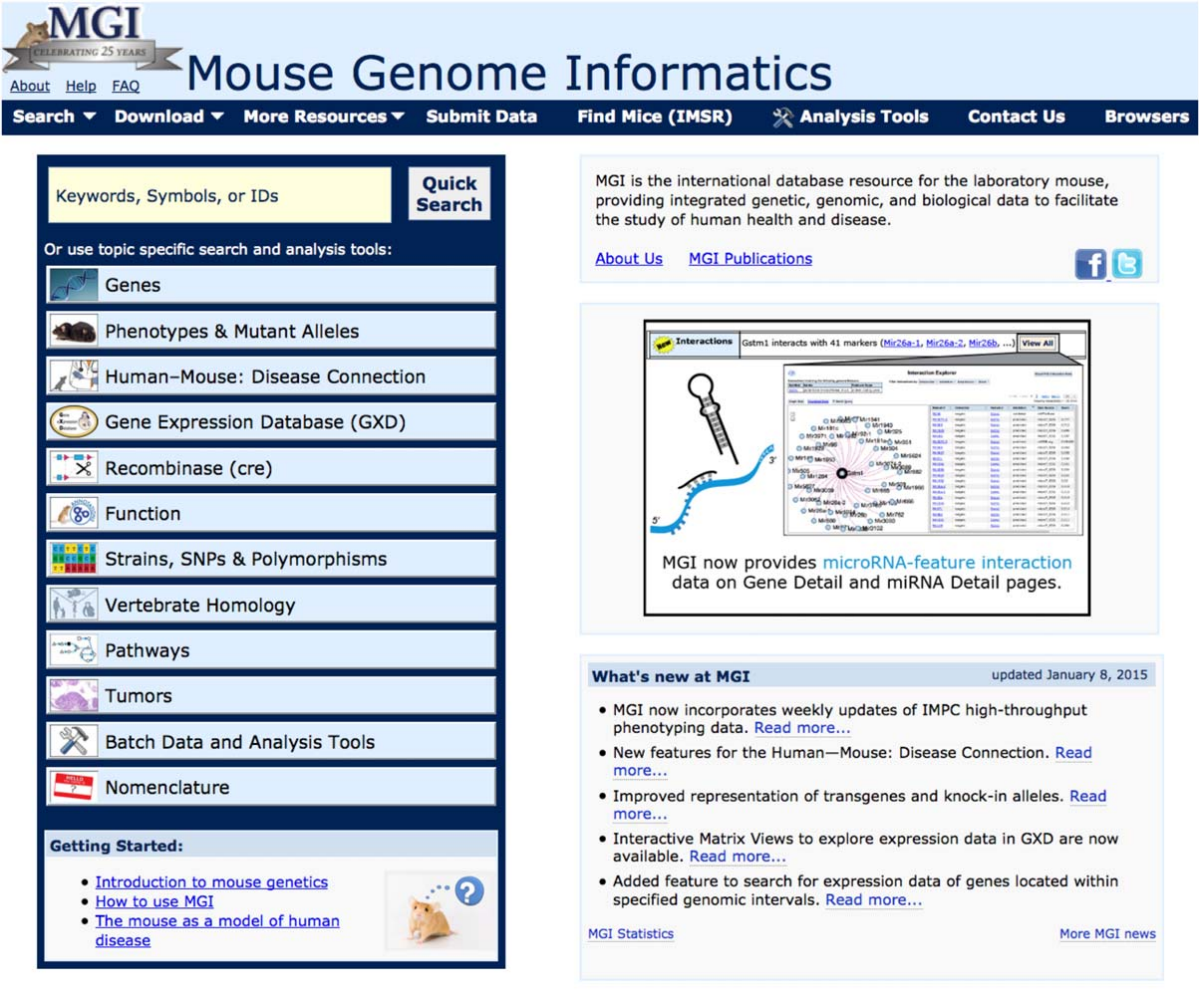
MGD Homepage. This figure shows the top portion of the homepage including the table of contents for the database. In the topic‐specific boxes (left), the major areas of data content are presented. Each topic‐specific box is a link to a sub‐page describing contents of that topic, links to specific search pages for that topic, FAQs, and links to documentation and collaborators. Beneath the topic index on the homepage is a “Getting Started” section of particular interest to new users. The “Quick Search” at the top of the table of contents is described further in Figure 3. The right side of the homepage includes links to and “About” page and to “MGI Publications,” a rotating informational image, a “What's New” section, providing information on the latest software and web changes, and MGI statistics on data content. The bottom section (not shown) holds items of “Community Interest.” The navy blue navigation bar at the top appears on each web page and allows users to quickly jump to the area of interest within MGD. The “Search,” “Download,” and “More Resources” items are each pull‐down menus leading to additional choices.

Users are invited to explore MGD at www.informatics.jax.org (Fig. 2). The homepage features access to the Quick Search tool, a popular starting point for many purposes. There is a menu of topic areas found at the Mouse Genome Informatics site, where one can explore specific interests, and a “Getting Started” section for new users. The navy blue navigation bar at the top is present on all web pages, making it easy to jump from one data area to another. In the navigation bar, the “Search,” “Download,” and “More Resources” choices have pull‐down menus as an additional navigation aid. Also available on the homepage is information about MGI publications, new features, and links to database statistics and former news. In the following sections of this review, we highlight key MGD features to provide a general flavor of the web interface. Users are encouraged to take advantage of our outreach and user help (see below) for assistance and questions about specific elements of the MGD web site.

### Quick Search Tool

**Figure 3:**
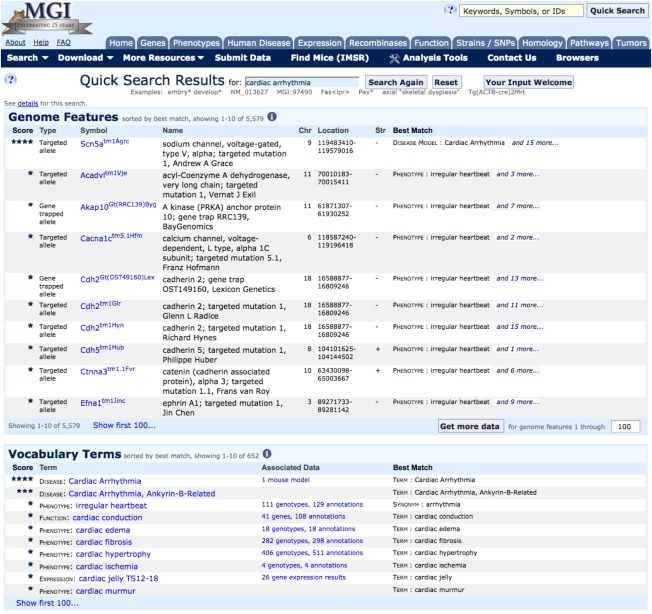
Results of a Quick Search query. In this example, the search term *cardiac arrhythmia* was used. The results page displays two sections: Genome Features, delineating genes and genome features matched in the search (10 displayed of 5,579 results that can be viewed sequentially) and Vocabulary Terms, showing various annotations in MGD to phenotype, disease, expression, function, and protein domain terms matched in the search (10 of 652 results shown). Results are returned by weighting, so that the “best matched” results appear first. Rarely does one need to scroll through many pages to discover their term of interest.

A primary access point into MGD is through use of the *Quick Search* tool, available from nearly every web page on the site (Fig. 3). The Quick Search tool is useful for those already familiar with MGD's web pages, who are looking to access a very specific piece of information quickly (e.g., find gene “X”). The Quick Search tool also provides a broad sweep across MGD data, providing an easy entry point for users unfamiliar with MGD content (e.g., what kinds of information are available on “apoptosis” or related to “cardiomyopathy”). The Quick Search tool provides a variety of links to follow for more details.

The Quick Search tool is located prominently on the homepage at the top of the topic index and on all other pages at the upper right corner. The Quick Search tool allows users to enter keywords, symbols, or IDs from multiple databases (e.g., RefSeq, Ensembl, PubMed, MGI). Results are returned as a “weighted list by best match” in two sections: (1) by Genome Feature, where the search targets nomenclature or annotations to genes or genome feature symbols, names, former symbols, allele symbols, synonyms, etc. and (2) by Vocabulary Terms, where the search targets data annotations made against specific vocabulary and ontology terms employed in MGD. In this case, the results returned will be one or more terms from the Mouse Developmental or Adult Anatomies, the Mammalian Phenotype Ontology, Gene Ontology, OMIM disease terms, and protein domains and families with links to the term and links to the annotated data.

### Advanced Search Forms

Depending on the search terms or IDs entered, the Quick Search tool can return thousands of results to sort through. In such cases, using more specific terms with the Quick Search tool or using one of the more targeted topic‐specific search forms that allows multi‐parameter searching can produce better results. For example, consider the results obtained with the Quick Search when entering the term *neuron*, which yields 9,218 Genome Feature matches and 1,122 Vocabulary matches (as of May 2015). The Genome Feature search results include both nomenclature matches to gene names and synonyms, as well as phenotype, disease, and functional annotations to the genome features. In contrast, entering the more specific term *“motor neuron degeneration”* (with quotes for exact match) yields 103 Genome Feature matches and 2 Vocabulary matches. Note that motor neuron degeneration *without* quotes will return a large number of results, as this quick search is performed on the entire term, motor neuron degeneration, as well as each word and each combination of words in the 3‐word string (motor, neuron, degeneration, motor neuron, motor degeneration, neuron degeneration).

**Figure 4:**
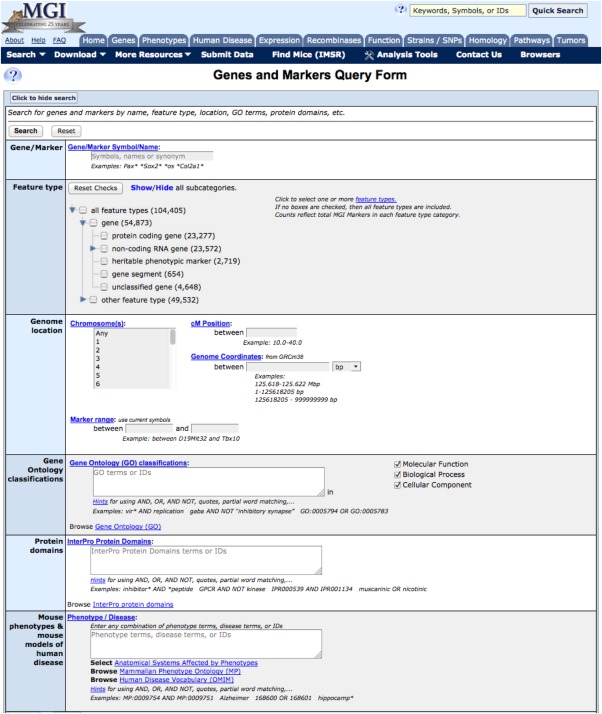
Genes and Marker Query Form. This Advanced Query Form illustrates the further precision in searching that one can obtain using specific Query Forms versus the Quick Search method. Here one can specify gene(s) by name or symbol, as one can in the Quick Search. But, in addition, data can be further specified by one or more characteristics—feature type, genome location, GO terms, Protein domains, and mouse phenotypes or human disease terms. To access the *Genes and Markers Query* form use the “Search” pull‐down menu in the navigation bar and follow the Genes section to select *Genes and Markers Query* or click the Gene topic box in the content boxes on the homepage and follow the appropriate link.

Using MGD's Advanced Search forms allows users to more precisely specify the parameters of a search and immediately narrow the scope of the search. The Advanced Search forms are accessed from any web page using the pull‐down menu under “Search” in the navy blue navigation bar at the top of the page and choosing a search form under the listed topics. For example, one can use the more targeted *Genes and Markers Query* form (Fig. 4), accessed under the “Search” pull‐down, submenu Genes. Once on the *Genes and Markers Query* form, entering the term *motor neuron degeneration* in the Gene/Marker box searches *only* MGD nomenclature and returns two genes where motor neuron degeneration is part of the name of mutant alleles of these genes.

### Mouse Genome Browser

**Figure 5:**
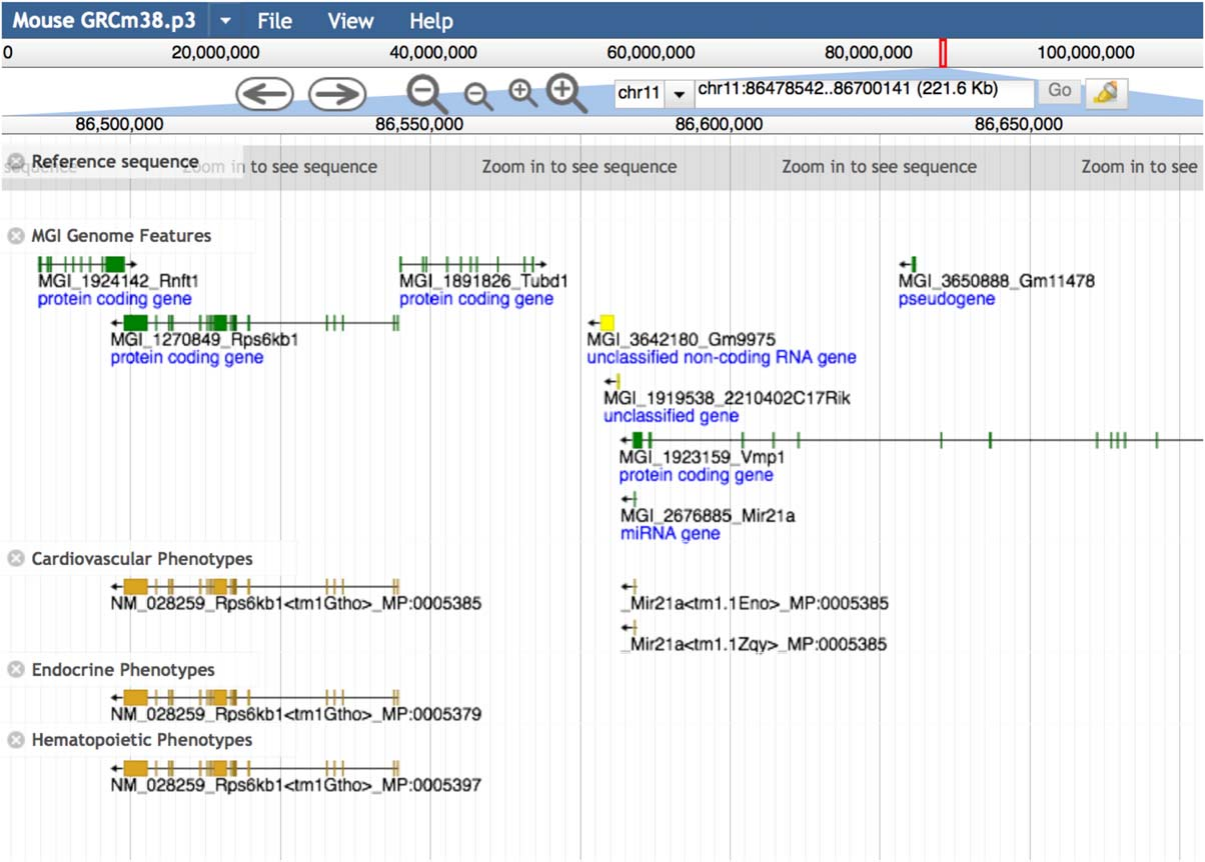
Mouse Genome Browser results. Here the Mouse Genome Browser is providing a view of 221.6 kb of chromosome 11 from nucleotide 86478542 to 86700141. Genome features are displayed at the top (green) gene structures and the associated phenotypes shown in gold. There are several ways to access the Mouse Genome Browser, including from the navigation bar of each web page under the “Search” pull‐down menu, where it is listed directly as the last item in the pull‐down. In addition, the Mouse Genome Browser link appears on “Search” submenus of the Genes or Sequences pull‐down menu items, and under the Genes topic box on the homepage.

MGD's Mouse Genome Browser is implemented using the open‐source genome browser tool, JBrowse (Skinner *et al*., 2009). JBrowse provides interactive web graphical displays of genome data and allows for frequent data updates and incorporation of large data files from MGD and external data sources. Currently, MGD's JBrowse implementation includes the following data types from MGD: genome features, alleles, phenotypes, QTL, and gene traps; from Ensembl: genes and regulatory features; from NCBI: genes and consensus CDS (cDNAs); and from Wellcome Trust Sanger Institute: Havana/Vega genes and Sanger SNPs. Also available for viewing is the human GRCh38 genome build and a pseudo genome for the CAST/EiJ mouse strain based on the analysis of sequence alignments (Munger *et al*., 2014). The integration of biological annotations with large‐scale sequence data and the mouse reference genome allow users to explore a genome view and biological context simultaneously. There are several ways to access the Mouse Genome Browser, including from the navigation bar of each web page under the “Search” pull‐down menu, where it is listed directly as the last item in the pull‐down. In addition, the Mouse Genome Browser link appears on “Search” submenus of the Genes or Sequences pull‐down menu items, and under the Genes topic box on the homepage (Fig. 5).

### Vocabulary/Ontology Browsers

As vocabularies and ontologies are key in mouse data annotation, MGD provides users with the ability to browse the terminologies. For simple vocabularies, such as OMIM terms, an alphabetical listing is provided with links from the disease term to the MGD Human Disease and Mouse Model Page, and from the OMIM ID to the disease entry at www.omim.org. For ontologies, including GO, Mammalian Phenotype, and Developmental and Adult Anatomies, the ontologies are presented hierarchically, reflecting the ontology structure and links are provided to all annotations associated with each term. These browsers are accessed via the navigation bar, under the Search pull‐down menu and the sub‐menu “Vocabularies.”

### Human‐Mouse: Disease Connection Interface

The Human‐Mouse: Disease Connection (HMDC, Eppig *et al*., 2015) is a translational tool developed to aid researchers in finding mouse models of human diseases and in identifying novel candidate disease genes. HMDC is accessed at www.diseasemodel.org, or by selecting “Human‐Mouse: Disease Connection” from the topic boxes on the MGD homepage, or using the “Search” pull‐down menu in the navigation bar and selecting “Human Disease.”

**Figure 6:**
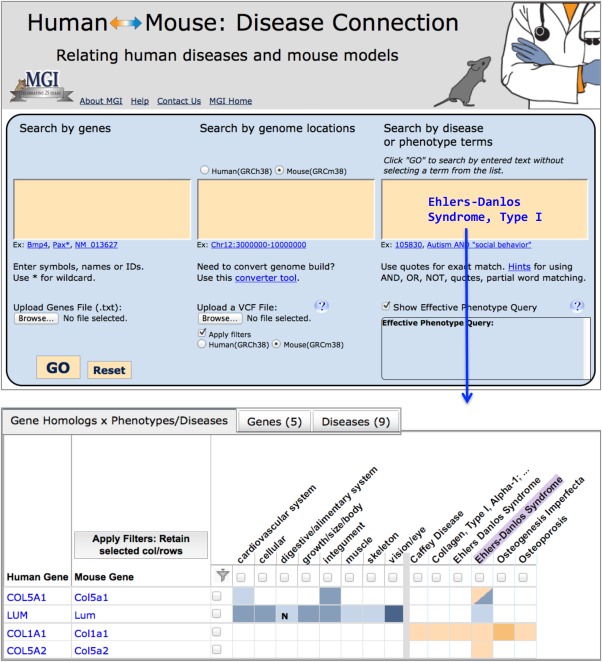
Human‐Mouse: Disease Connection (HMDC), www.diseasemodel.org. The top panel shows the upper portion of the HMDC homepage with three distinct search boxes to allow searching by either mouse or human genes, genome locations, or disease or phenotype terms. Note that options are provided to upload a gene file or a VCF file to use as search parameters as well. In this example Ehlers–Danlos syndrome, Type I was entered in the disease/phenotype term box. The lower panel shows the resulting grid display where human and mouse orthologs are shown in rows and phenotypes and diseases are shown in columns. Blue indicates mouse data; orange indicates human data. The highlighted Ehlers–Danlos syndrome column shows both human *COL5A1* and mouse *Col5a1*, respectively, are associated to the disease. Mouse gene *Lum* and human genes *COL1A1* and *COL5A2* are associated to this human disease as well, but not coincidentally. These data suggest that mice with mutations in *Col1a1* and *Col5a2* should be examined for phenotypes correlated to human Ehlers–Danlos syndrome and that human patients with Ehlers–Danlos phenotypes might be checked for mutations in the *LUM* gene.

Taking advantage of MGD's integrated mouse and human genomic, phenotypic, and disease data, users can approach the HMDC from a human or mouse perspective, searching for symbols, names, or IDs for genes, genome locations, or disease or phenotype terms. VCF (variant call format) files and text files of genes or gene IDs may be used as input as well. The initial search returns a grid (Fig. 6) with rows of human and mouse orthologs, and columns of phenotypes and human disease terms that are color‐coded (blue for mouse data; orange for human data). Important in this example of Ehlers–Danlos syndrome, Type I, is that the ortholog pair human *COL5A1*and mouse *Col5a1* genes are both known to be associated with this disease as denoted by the intersecting bi‐colored grid cell. Ehlers–Danlos syndrome, Type I is also associated with human genes *COL1A1* and *COL5A2* as denoted by orange grid cells. The mouse orthologs of both of these genes (*Col1a1* and *Col5a2*) have known phenotypic mutations in MGD, and thus are ready‐made candidate genes for experimental validation as new Ehlers–Danlos syndrome, Type I models. Conversely, there is a mouse model for Ehlers–Danlos syndrome, Type I with mutations in mouse gene *Lum* (lumican), but the human ortholog has not yet been associated with this disease. Thus human *LUM* may be a new candidate to consider as causative in human patients or may be in a critical pathway for Ehlers–Danlos syndrome. Each colored grid cell is an active link to additional data detail, leading to specific genotype information, MP ontology annotations, links to obtaining mouse models, references, and more. Other data views are seen using the “Genes” and “Diseases” tabs located above the grid.

## WORKING WITH LARGE DATA SETS IN MGD

### Downloading MGD Data

Many web pages throughout the MGD website provide the ability to download the current data being viewed in text or Excel format. In addition, MGD maintains a large set of text files with specific subsets of data on the FTP site. These are frequently downloaded by collaborators and other biomedical resources that regularly incorporate MGD data, as well as by computational users. The files are updated weekly. Further, complete database dumps are accessible, along with a schema browser. A link to an index of commonly used FTP files (All MGI Reports) arranged by topic area with a description of their file contents and download link can be found in the “Download” pull‐down menu on the navigation bar at the top of all MGD web pages. Users also can select a specific topic area to view subsets of MGD data reports or select Batch Query to create their own unique reports. Additional resources can be found using the “More Resources” pull‐down on the navigation bar and selecting More Resources Index.

### Batch Query

Users often come to MGD with a set of genes of interest and the desire to retrieve additional information about that set of genes. The MGD Batch Query tool (Bult *et al*., 2008) allows the user to type in, paste, or upload thousands of gene symbols or IDs from various data sources (MGI, Ensembl, RefSeq, etc.) and specify the additional information desired (GO annotations, mutant alleles, MP annotations, RefSNP IDs, Human Disease annotations, etc.). The Batch Query tool also serves as a convenient “ID converter,” so that, for example a set of MGI IDs can bring back IDs for the same genes in Ensembl, NCBI Gene, and VEGA. The results are displayed as a paginated table with links to underlying data in MGD and the results table can be downloaded. The Batch Query tool can be found from the homepage by (1) selecting “Batch Data and Analysis Tools” in the topic selection boxes or, (2) using the navigation bar at the top of the page and selecting Batch Query from the “Search” pull‐down menu or from the “Download” pull‐down menu or following the “Analysis Tools” link in the bar.

### MouseMine

MouseMine (Sullivan *et al*., 2013) is the mouse‐specific instance of InterMine (Kalderimis *et al*., 2014), an open source software framework supporting data warehousing with an interface of powerful tools for data querying, retrieval, and analysis. Users can access Mines of other Model Organisms (MODs) using common interfaces and tools, thus greatly lowering the barrier to exploring data in different MODs (see Lyne *et al*., 2015 for an extensive review of InterMine). MouseMine is accessed via www.mousemine.org, by selecting MouseMine from the “Search” pull‐down menu on the MGD web page navigation bar or by selecting “Batch Data and Analysis Tools” from the topic boxes on the homepage.

MouseMine includes most of the core data present in MGD (e.g., genes, genome features, genome coordinates, alleles and mutations, mouse strains and genotypes, ontologies, functional annotations, phenotype annotations, human disease annotations, vertebrate homologies, references, etc.), Data are updated weekly from MGD. In addition, other data sets not currently included in MGD are integrated in MouseMine through common object identifiers, such as homology data from PANTHER (Protein Analysis THrough Evolutionary Relationships, Mi *et al*., 2010).

The MouseMine interface is not as user friendly as the MGD web interface, but provides a powerful tool for developing specific database queries that are not addressed using the MGD web query forms. Thus, MouseMine is a highly valuable tool for knowledgeable users and computational biologists. With MouseMine complex queries can be specified and display content can be individualized. Users can also save or download results for future analysis, export results to other tools such as Galaxy (Goecks *et al*., 2010) or Genomespace (http://www.genomespace.org), or apply MouseMine's enrichment analysis tools. As well, MouseMine also provides a RESTful web services API (Applications Program Interface).

## COMMUNITY: OUTREACH AND USER SUPPORT

### User Input and Data Submission

MGD encourages user contributions in many ways. On each Gene Detail page and Allele Detail page, a “Your Input Welcome” button appears where users can comment on the page they are viewing by contributing new data or references or commenting if an error is noticed.

A suite of data submission tools is provided by clicking the “Submit Data” option in the MGD navigation bar at the top of each web page. These tools allow investigators to submit a new locus for establishing official nomenclature, submit a new variant or mutant allele, register a new mouse strain or submit phenotype data or expression data. Investigators with large or specialized data sets can contact MGD for assistance in formatting and contributing their data.

### User Support

MGD provides a variety of User Support services. Each MGD web page includes a “Contact Us” option on the navigation bar that leads to a web form for comments and questions. An active email address, mgi-help@jax.org is monitored 5‐days per week for questions regarding data content or nomenclature, data access, bug reports, and data submission. User Support staff maintain an active community Listserve with nearly 2,000 active users participating in forum discussions.

### Online Help

MGD maintains extensive online documentation and tutorials covering current data content and interfaces. These include information about what kinds of data are integrated into MGD, how those data are annotated, help with searching, and definitions and explanations of the fields displayed in search results. In addition, Frequently Asked Questions (FAQ) pages provide illustrated examples that walk the user through common searches (e.g., How do I search for a gene in a specific genomic interval?). Links to “Help” and “FAQ” pages are located on all web pages in the upper left corner beneath the MGI logo. The homepage also includes a “Getting Started” section located beneath the topic index in the left column (Fig. 2). Newly added features and significant data content changes are highlighted in the “News and Release Announcements” section of the homepage.

### Digital Media

MGD is on Facebook (www.facebook.com/mgi.informatics) and maintains a Twitter account (MouseGenomeInfo@mgi_mouse). A newly released iPhone app, MGI GenomeCompass, includes the ability to save “favorite” genes, phenotypes, or diseases, allowing the user to receive automated updates whenever they log in. An Android app is in the planning stage.

### MGD Workshops

MGD staff frequently provide individual or group tutorials in conjunction with courses and conferences at the Jackson Laboratory and provide off‐site workshops at various institutions as feasible. When MGD staff attend off‐site conferences, these can be coupled with local workshops or MGD staff can schedule customized workshops on request (see http://www.informatics.jax.org/mgihome/support/Roadshow_description.pdf).

## EVOLUTION AND ONGOING CHALLENGES FOR MGD

The issues of growing and maintaining a viable bioinformatics resource for the research community are complex. MGD plays a critical role in the ecosystem of major biological databases. To maintain its necessary rapid and continued growth in data size and changing data types means continually searching for new efficiencies on every level, evaluating trends (what data types are no longer relevant? what are the next challenges from new biological data types), and reaching for new emerging collaborations.

Some clear examples of MGD's evolution include:

1. At the beginning, genes were placed on linkage maps; there were no sequences, only a few RFLPs (restriction fragment length polymorphisms); we did not know how many genes there might be; we sought to provide better maps, and identify new mutations that might help (indirectly) find new genes. As the Human Genome Project gathered momentum and the mouse began to be sequenced in earnest, attention turned to sequencing (still painfully slow and difficult to analyze); but grew in capability and analytical tool collections. MGD moved first to support physical maps and radiation hybrid maps, and finally to contig and sequence maps.
2. Mutations were initially detected by observation of spontaneous visible phenotypes. Radiation and certain mutagens began to be used for mutation induction, though their mechanisms were poorly understood. Later studies using ENU mutagenesis, where dosage was studied and mutagenesis maximized, became a standard and generated thousands of new mutations for study. Genetic engineering in mice became possible and propelled mice into a wunderkind position with this unique and powerful tool that remains a mainstay of mutagenesis and enables highly innovative and specifically designed genetic changes.
3. Sequencing technology continues to get cheaper, and with it the ability to sequence multiple inbred strains for variant analysis and to quickly identify the precise DNA change(s) responsible for a mutation, whether spontaneous or induced. We soon will be sequencing all our experimental animals and have individual profiles for each mouse.

The above are punctuated paradigm shifts brought about by adoption of new biological technology at a community scale, which continues to flourish and produce innovation for tomorrow's research. The database resources in this issue of *Genesis* represent those that have been able to adapt to the kinds of major research shifts mentioned above, as well as being adaptive to other disruptive advances in computer technologies necessary to provide the infrastructure for continued database advances.

## Footnotes

1 The Gene Trap Consortium is no longer generating new mutations, but ES cell lines from this effort are available to researchers. See the International Strain Resource (www.findmice.org).

2 To perform this search, enter “Tap” in the Quick Search tool or the Genes and Marker Query form. Search results indicate Tap as a synonym in the right‐hand column. See additional detail in later sections of this review on the Quick Search and Advanced Search Forms.
